# Genistein Alleviates Intestinal Oxidative Stress by Activating the Nrf2 Signaling Pathway in IPEC-J2 Cells

**DOI:** 10.3390/vetsci11040154

**Published:** 2024-03-29

**Authors:** Yanpin Li, Long Cai, Qingyue Bi, Wenjuan Sun, Yu Pi, Xianren Jiang, Xilong Li

**Affiliations:** 1 Key Laboratory of Feed Biotechnology of Ministry of Agriculture and Rural Affairs, Institute of Feed Research, Chinese Academy of Agricultural Sciences, Beijing 100081, China; liyanpin@caas.cn (Y.L.); 82101211211@caas.cn (L.C.); biqingyue981106@126.com (Q.B.); sunwenjuan@caas.cn (W.S.); jiangxianren@caas.cn (X.J.); 2 College of Agriculture, Yanbian University, Yanji 133000, China

**Keywords:** genistein, antioxidation, Nrf2 signaling pathway, intestinal epithelial cells, piglets

## Abstract

**Simple Summary:**

Interest in using natural feed additives in animal diets to improve health and productivity has grown significantly over the past few decades. Genistein is an aglycone form of soybean isoflavones with higher antioxidant activity. However, whether genistein can alleviate oxidative stress in pig intestines and the precise mechanism behind this effect remains to be elucidated. In the present research, the hydrogen peroxide-stimulated IPEC-J2 cells oxidative stress model was employed to explore the antioxidant capacity of genistein and potential mechanisms. The results showed that genistein could exert a protective effect against hydrogen peroxide-stimulated oxidative stress by activating the Nrf2 signaling pathway in IPEC-J2 cells. These results could provide a novel nutritional intervention strategy to enhance the intestinal health of piglets under oxidative stress.

**Abstract:**

In the weaning period, piglets often face oxidative stress, which will cause increased diarrhea and mortality. Genistein, a flavonoid, which is extracted from leguminous plants, possesses anti-inflammatory and antioxidative bioactivities. However, little is known about whether genistein could attenuate the oxidative stress that occurs in porcine intestinal epithelial cells (IPEC-J2). Herein, this experiment was carried out to investigate the protective effects of genistein in the IPEC-J2 cells oxidative stress model. Our results disclosed that H_2_O_2_ stimulation brought about a significant diminution in catalase (CAT) activity and cell viability, as well as an increase in the levels of reactive oxygen species (ROS) in IPEC-J2 cells (*p* < 0.05), whereas pretreating cells with genistein before H_2_O_2_ exposure helped to alleviate the reduction in CAT activity and cell viability (*p* < 0.05) and the raise in the levels of ROS (*p* = 0.061) caused by H_2_O_2_. Furthermore, H_2_O_2_ stimulation of IPEC-J2 cells remarkably suppressed gene level *Nrf2* and *CAT* expression, in addition to protein level Nrf2 expression, but pretreating cells with genistein reversed this change (*p* < 0.05). Moreover, genistein pretreatment prevented the downregulation of occludin expression at the gene and protein level, and *ZO-1* expression at gene level (*p* < 0.05). In summary, our findings indicate that genistein possesses an antioxidant capacity in IPEC-J2 cells which is effective against oxidative stress; the potential mechanism may involve the Nrf2 signaling pathway. Our findings could offer a novel nutritional intervention strategy to enhance the intestinal health of piglets during the weaning process.

## 1. Introduction

Oxidative stress arises from an imbalance within the oxidative and antioxidant systems and is related to an excessive yield of reactive oxygen species (ROS) [1]. It damages proteins, lipids, and DNA, resulting in tissue injury, cell death, and eventually causes the development of certain diseases [2]. The intestine is not only the main organ for digestion and absorption of nutrients, but it also acts as the most critical barrier against harmful pathogens, toxins, and antigens from the luminal environment [3,4]. However, as the boundary between the body and the environment, the intestine is more susceptible to oxidative stress due to its sustained exposure to the complex physiological or chemical environment [5,6]. Thus, protecting intestinal epithelial cells from oxidative stress is critical to the intestinal function, growth, and health of animals.

Based on the findings of our former research, it appears that soybean isoflavones play a crucial role in maintaining optimal antioxidant capacity and growth in pigs [7]. Excluding soybean isoflavones from their diet led to a reduction in growth performance and antioxidant properties, whereas re-adding soybean isoflavones prevented these negative effects [7]. However, soybean isoflavones in diets exist mainly in the form of glycosides, which are transformed into aglycones through deglycosylation under the action of enzymes in the intestine, and the aglycones are then absorbed by the intestine and circulated throughout the body [8,9]. In addition, the structure of glycosides is relatively complex and usually obtained by extraction, whereas the structure of aglycones is relatively simple and can be obtained by chemical synthesis, which not only significantly improves the purity but also greatly reduces the cost. Thus, we further investigated the application effect of daidzein (4′,7-dihydroxyisoflavone, an aglycone form of the soybean isoflavones) supplementation in basal diets containing soybean meal on weaned piglets, and the results showed that the basal diet added with daidzein (50 mg/kg) could efficaciously enhance antioxidant capacity and growth in weaned piglets [10]. Moreover, daidzein exerted protective effects in the porcine intestinal epithelial cells (IPEC-J2) oxidative stress model, and the underlying mechanism was possibly associated with activating the Nrf2 signaling pathway [10]. Genistein (4′,5,7-trihydroxyisoflavone), another main aglycone form of the soybean isoflavones, possess multiple bioeffects, including antioxidant, anticancer, and anti-inflammatory properties [11,12,13]. Several studies have reported the antioxidant properties of genistein [14,15]. However, only a limited number have explored the antioxidant properties of genistein, thus there is a lack of understanding regarding its positive effects on the alleviation of intestinal oxidative stress in pigs.

Therefore, the aim of our research was to estimate the antioxidant capacity and the potential mechanisms of genistein in IPEC-J2 cells. Our results provide insights for future applications of genistein as an antioxidant against intestinal oxidative stress in the pig industry.

## 2. Materials and Methods

### 2.1. Reagents

DMEM/F12, penicillin-streptomycin, fetal bovine serum, TRIzol reagent, SYBR Green, and RIPA buffer were supplied from Thermo Fisher Scientific (Waltham, MA, USA). Epidermal growth factor, and ITS (the mixture of insulin, transferrin, and selenious acids) were obtained from Corning Incorporated (New York, NY, USA), dimethyl sulfoxide, and genistein (synthetic product, purity ≥ 98%) were provided by Sigma-Aldrich (St. Louis, MO, USA). ROS assay kit was obtained from Beyotime Biotechnology (Shanghai, China). The cell counting kit (CCK-8) was bought from Med Chem Expression (Princeton, NJ, USA). The malondialdehyde (MDA), glutathione peroxidase (GSH-Px), catalase (CAT), and superoxide dismutase (SOD) assay kits were provided by Nanjing Jiancheng Bioengineering Institute (Nanjing, China). The Trans Script First-Strand cDNA Synthesis Kit was supplied by Trans Gen Biotech (Beijing, China). The VDF membranes, and ECL agent were provided by Bio-Rad Laboratories, Incorporated (Irvine, CA, USA).

### 2.2. Cell Culture

We thank the laboratory of Dr. Guoyao Wu at Texas A&M University for providing the IPEC-J2 cells, a well-established non-transformed porcine intestinal epithelial cell line derived from neonatal piglets’ mid-jejunum [16]. DMEM/F12 medium added with 0.01% epidermal growth factor, 0.1% ITS and 1% penicillin-streptomycin, and 5% fetal bovine serum was used for culture. The cells were cultured in a humidified incubator with 5% CO_2_ at 37 °C.

### 2.3. Selection of Genistein Concentration

The concentration (10 mg/mL) of genistein was dissolved using dimethyl sulfoxide. The 96-well plate was used to seed the IPEC-J2 cells with 1 × 10^4^ cells per well for 24 h. Afterwards, the cells were pretreated with diverse concentrations of genistein (0, 10, 20, 40, 60, and 80 μM) for an additional 24 h, followed by treatment with or without 0.6 mM H_2_O_2_ for a further 1 h. The concentrations and treatment times of H_2_O_2_ are based on our prior research [10]. The CCK-8 kit was used to measure cell viability following the manufacturer’s instructions. In brief, following incubation for 3 h at 37 °C with 10% CCK-8 reagent, cell absorbance was recorded at 450 nm. The formula for calculating cell viability is as follows: Cell viability = [(the absorbance of the treatment group) − (the absorbance of the blank group)]/[(the absorbance of the control group) − (the absorbance of the blank group)] × 100%. We considered the control group’s cell viability to be 100%.

### 2.4. Determination of Intracellular ROS

The 96-well plate was used to seed the IPEC-J2 cells with 1 × 10^4^ cells per well for 24 h. Then, the medium was changed with or without 20 μM genistein for an additional 24 h, followed by treating the cells with or without 0.6 mM H_2_O_2_ for another 1 h. The ROS assay kit was used to test the intracellular ROS levels based on the manufacturer’s instructions. The cells were cultured with DCFH-DA probes for 30 min, then washed 3 times using PBS. Subsequently, the fluorescence was recorded at 525 nm and 488 nm for emission and excitation, respectively.

### 2.5. Determination of Antioxidant Indices

The 6-well plate was used to seed the IPEC-J2 cells with 3 × 10^5^ cells per well for 24 h. Then, the medium was changed with or without 20 μM genistein for an additional 24 h, followed by treatment of the cells with or without 0.6 mM H_2_O_2_ for a further 1 h. For subsequent lysis, we used RIPA buffer for 30 min, and centrifuged the supernatant for 30 min at 13,000× *g*. Then, we determined the malondialdehyde (MDA) levels, and CAT, SOD, GSH-Px activities using the MDA, CAT, SOD and GSH-Px assay kits as directed by the manufacturer. Briefly, MDA concentration was analyzed with 2-thiobarbituric acid, and the change in absorbance was read at 532 nm. CAT activity was measured with ammonium molybdate, and the change in absorbance was recorded at 405 nm. SOD activity was measured through a nonenzymatic NBT test, which measures the inhibition of the formation of superoxide anion free radicals that reduce the nitroblue tetrazolium of the sample. The change in absorbance at 450 nm was recorded. GSH-Px activity was measured with 5,50-dithiobis-p-nitrobenzoic acid, and the change in absorbance at 412 nm was recorded.

### 2.6. RNA Extraction, Reverse Transcription, and Quantitative Real-Time PCR (qPCR)

The 12-well plate was used to seed the IPEC-J2 cell with 1.5 × 10^5^ cells per well for 24 h. Following this, the medium was changed with or without 20 μM genistein for an additional 24 h, followed by treatment of the cells with or without 0.6 mM H_2_O_2_ for another 1 h. TRIzol reagent was used to extract the total RNA from the cells according to the manufacturer’s instructions. The concentration and A260/A280 ratio of the total RNA was determined using an Epoch microplate spectrophotometer. An A260/280 ratio ranging between 1.8 and 2.0 was subjected to further analysis. Total RNA (1 μg) was reverse transcribed into cDNA using a Trans Script First-Strand cDNA Synthesis Kit. SYBR Green PCR Master Mix was used to perform qPCR analysis in the CFX96 Real-Time System. The qPCR reaction system was: 2 μL cDNA template, 0.5 μL forward primer, 0.5 μL reverse primer, 10 μL SYBR Green PCR Master Mix, and 7 μL DNase and RNase free water. The reaction procedure was as follows: denature: 50 °C for 2 min, 95 °C for 10 min; extension: 95 °C for 15 s, 60 °C for 1 min, 40 cycles; and melt curve: 95 °C for 15 s, 60 °C for 1 min, 95 °C for 15 s. The relative expressions of all the target genes were calculated using the 2^−ΔΔCT^ method [17] and the housekeeping gene was glyceraldehyde-3-phosphate dehydrogenase (*GAPDH*). Table 1 presents the sequences of primers.

### 2.7. Western Blotting

The 6-well plates were used to seed the IPEC-J2 cell with 3 × 10^5^ cells per well for 24 h. Then, the medium was changed with or without 20 μM genistein for an additional 24 h, followed by treatment of the cells with or without 0.6 mM H_2_O_2_ for a further 1 h. For subsequent lysis, we used RIPA buffer for 30 min, and centrifuged the supernatant for 30 min at 13,000× *g*. The samples were transferred to PVDF membranes after being separated by the SDS-PAGE (12%). 5% skim milk in Tris-buffered saline with Tween 20 was used to block PVDF membranes at room temperature for 3 h, followed by overnight incubation at 4 °C in the presence of primary antibodies, and finally, they were incubated at 4 °C with secondary antibodies for 1 h. The ECL agent was used to detect the chemiluminescence signals, which were then visualized using the ChemiDoc MP Imaging System. The band intensity was quantified using Image J software (v1.8.0). The band of GAPDH was considered as the internal reference band. Table 2 lists detailed information on antibodies.

### 2.8. Statistical Analysis

Data analysis was performed using SPSS 20.0 software with the one-way ANOVA procedure. Treatment differences were assessed using Tukey’s post-hoc test. *p* < 0.05 was considered statistically significant, whereas 0.05 ≤ *p* < 0.10 indicated a trending treatment effect.

## 3. Results

### 3.1. Protective Effect of Genistein on Cell Viability

**Figure 1:**
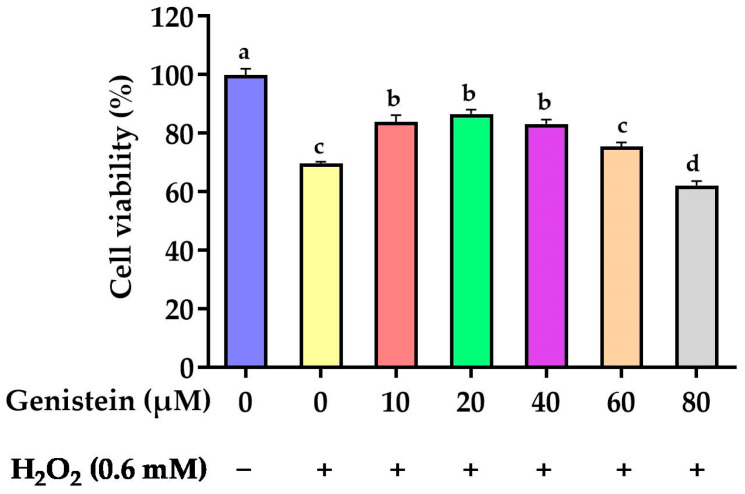
Effect of genistein on the IPEC-J2 cells viability. The 96-well plate was used to seed the IPEC-J2 cells, then the medium was changed with various genistein concentrations for 24 h, followed by the treatment of cells with or without 0.6 mM H_2_O_2_ for 1 h. Values are means ± standard error, *n* = 6. There are statistically significant differences (*p* < 0.05) between the means assigned the letters a–d.

As revealed in Figure 1, cell viability was significantly reduced from 100% to 69.8% in the H_2_O_2_-treated group compared to the control group (*p* < 0.05). However, in contrast to the H_2_O_2_-treated group, pretreating cells with 10, 20, and 40 μM genistein before H_2_O_2_ exposure enhanced cell viability from 69.8% to 83.8%, 86.5%, and 83.2%, respectively (*p* > 0.05). For subsequent experiments, we opted for a concentration of 20 μM genistein as it exhibited enhanced cell viability at this particular dosage.

### 3.2. Intracellular ROS Levels

**Figure 2:**
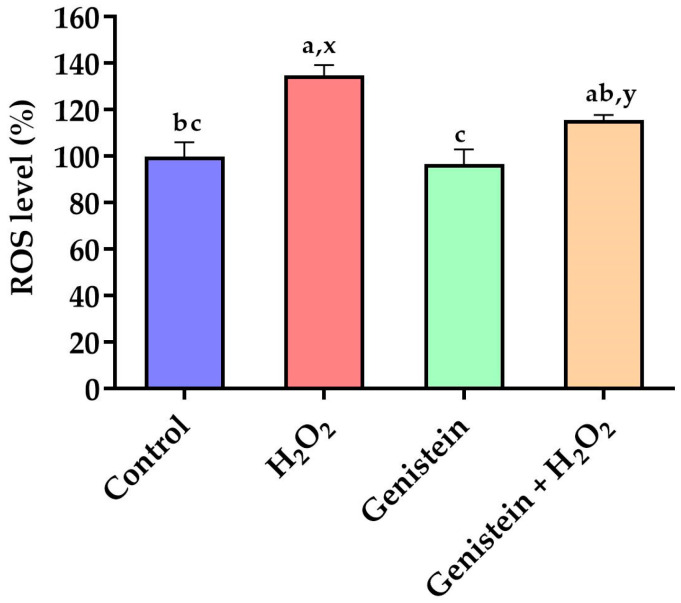
Effect of genistein on ROS levels in IPEC-J2 cells. The 96-well plate was used to seed the IPEC-J2 cells, then the medium was changed with or without 20 μM genistein for 24 h, followed by the treatment of cells with or without 0.6 mM H_2_O_2_ for 1 h, and finally, the cells were cultured with DCFH-DA probes for 30 min. Values are means ± standard error, *n* = 6. There are statistically significant differences (*p* < 0.05) between the means assigned the letters a–c; and there is a tendency (0.05 ≤ *p* < 0.10) between the means assigned the letters x, y.

As demonstrated in Figure 2, a significant elevation in intracellular ROS levels was observed in the H_2_O_2_-treated group compared to the control group (*p* < 0.05). In contrast to the H_2_O_2_-treated group, pretreating cells with 20 μM genistein before H_2_O_2_ exposure showed a tendency to reduce intracellular ROS levels (*p* = 0.061). Comparatively, the genistein treated group did not have elevated intracellular ROS levels (*p* > 0.05).

### 3.3. Antioxidant Enzyme Activities and MDA Level

**Figure 3:**
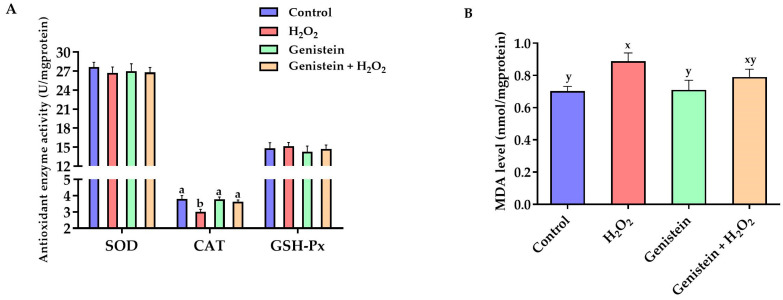
Effects of genistein on MDA level and antioxidant enzyme activities in IPEC-J2 cells. (**A**) Antioxidant enzyme activities; (**B**) MDA level. The 6-well plate was used to seed the IPEC-J2 cells, then the medium was changed with or without 20 μM genistein for 24 h, followed by the treatment of cells with or without 0.6 mM H_2_O_2_ for 1 h. Values are means ± standard error, *n* = 6. There are statistically significant differences (*p* < 0.05) between the means assigned the letters a, b; and there is a tendency (0.05 ≤ *p* < 0.10) between the means assigned the letters x, y.

According to Figure 3, H_2_O_2_ stimulation significantly reduced the CAT activity (*p* < 0.05) and showed a tendency to increase the MDA level (*p* = 0.06) compared to the control group. However, in contrast to the H_2_O_2_-treated group, pretreating cells with 20 μM genistein before H_2_O_2_ exposure significantly elevated the CAT activity (*p* < 0.05). No significant differences in GSH-Px and SOD activities were observed (*p* > 0.05).

### 3.4. Expression of Key Genes in Nrf2 Signaling Pathway

**Figure 4:**
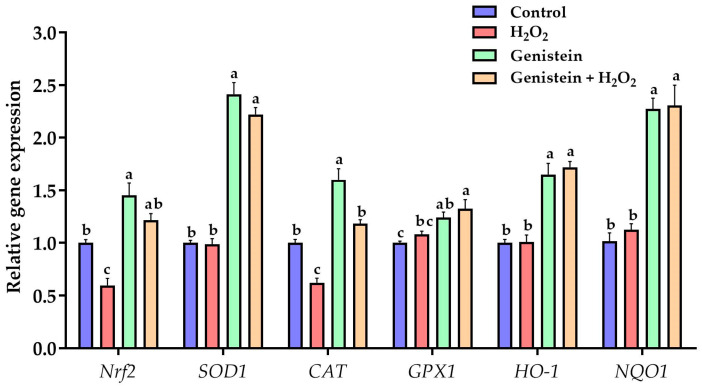
Effect of genistein on the expression of key genes in the Nrf2 signaling pathway in IPEC-J2 cells. The 12-well plate was used to seed the IPEC-J2 cells, then the medium was changed with or without 20 μM genistein for 24 h, followed by the treatment of cells with or without 0.6 mM H_2_O_2_ for 1 h. Values are means ± standard error, *n* = 6. There are statistically significant differences (*p* < 0.05) between the means assigned the letters a–c.

As presented in Figure 4, a significant reduction in the gene expression of *Nrf2* and *CAT* was observed in the H_2_O_2_-treated group compared to the control group (*p* < 0.05). However, in contrast to the H_2_O_2_-treated group, pretreating cells with 20 μM genistein before H_2_O_2_ exposure evidently elevated the gene expression of *SOD1*, *Nrf2*, *CAT*, *GPX1*, *NQO1*, and *HO-1*. In addition, a significant increase in the gene expression of *SOD1*, *CAT*, *Nrf2*, *NQO1*, *GPX1*, and *HO-1* was observed in the genistein treated group compared to the control group (*p* < 0.05).

### 3.5. Tight Junction Gene Expression

**Figure 5:**
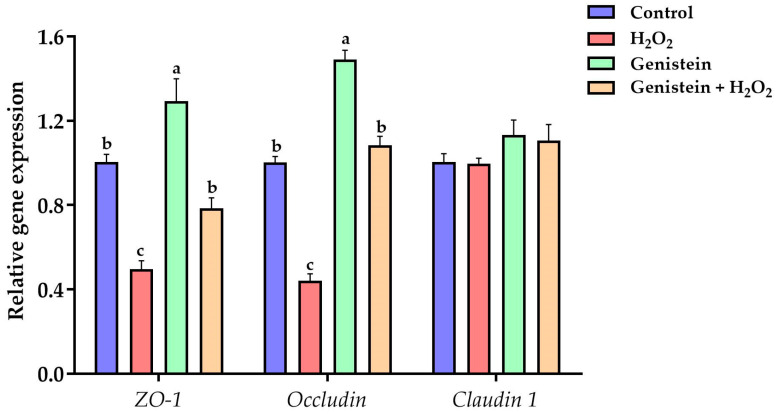
Effect of genistein on the tight junction gene expression in H_2_O_2_-treated IPEC-J2 cells. The 12-well plate was used to seed the IPEC-J2 cells, then the medium was changed with or without 20 μM genistein for 24 h, followed by the treatment of cells with or without 0.6 mM H_2_O_2_ for 1 h. Values are means ± standard error, *n* = 6. There are statistically significant differences (*p* < 0.05) between means assigned the letters a–c.

As displayed in Figure 5, a remarkable reduction in the gene expression of *occludin* and *ZO-1* was observed in the H_2_O_2_-treated group compared to the control group (*p* < 0.05). However, in contrast to the H_2_O_2_-treated group, pretreating cells with 20 μM genistein before H_2_O_2_ exposure led to a significant increase in the gene expression of *occludin* and *ZO-1* (*p* < 0.05). In addition, the gene expression of *ZO-1* and *occludin* in the genistein treated group was significantly increased in contrast to the control group (*p* < 0.05). Whereas, no significant differences in the gene expression of *claudin 1* were observed (*p* > 0.05).

### 3.6. Nrf2 Protein Expression

**Figure 6:**
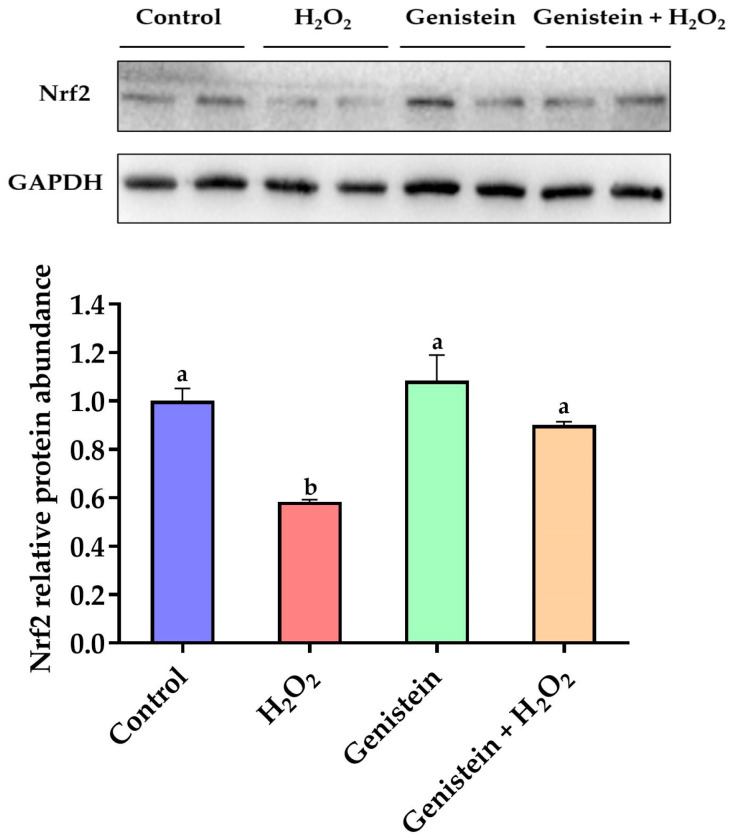
Effect of genistein on the Nrf2 protein expression in IPEC-J2 cells. The 6-well plate was used to seed the IPEC-J2 cells, then the medium was changed with or without 20 μM genistein for 24 h, followed by the treatment of cells with or without 0.6 mM H_2_O_2_ for 1 h. Values are means ± standard error, *n* = 4. There are statistically significant differences (*p* < 0.05) between means assigned the letters a, b. (Figures S1 and S2).

According to Figure 6, a remarkable reduction in the relative protein abundance of Nrf2 was observed in the H_2_O_2_-treated group compared to the control group (*p* < 0.05). However, in contrast to the H_2_O_2_-treated group, pretreating cells with 20 μM genistein before H_2_O_2_ exposure led to a significant increase in the relative protein abundance of Nrf2 (*p* < 0.05). Comparatively, the genistein treated group did not display the elevated relative protein abundance of Nrf2 (*p* > 0.05).

### 3.7. Tight Junction Protein Expression

**Figure 7:**
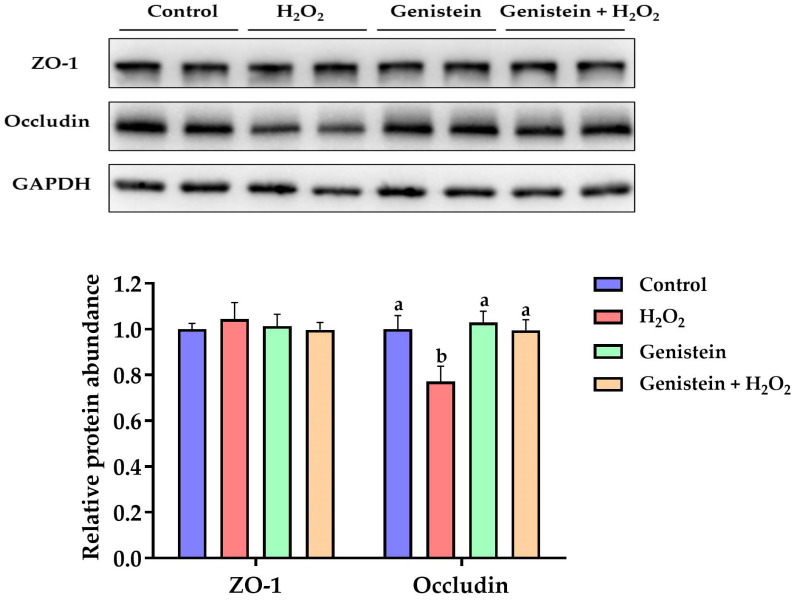
Effect of genistein on the tight junction protein expression in H_2_O_2_-treated IPEC-J2 cells. The 6-well plate was used to seed the IPEC-J2 cells, then the medium was changed with or without 20 μM genistein for 24 h, followed by the treatment of cells with or without 0.6 mM H_2_O_2_ for 1 h. Values are means ± standard error, *n* = 4. There are statistically significant differences (*p* < 0.05) between means assigned the letters a, b. (Figures S3 and S4).

As presented in Figure 7, a remarkable reduction in the relative protein abundance of occludin was observed in the H_2_O_2_-treated group compared to the control group (*p* < 0.05). However, in contrast to the H_2_O_2_-treated group, pretreating cells with 20 μM genistein before H_2_O_2_ exposure led to a significant increase in the relative protein abundance of occludin (*p* < 0.05). On the other hand, compared to the control group, the relative protein abundance of occludin in the genistein treatment group was not affected (*p* > 0.05). Furthermore, no significant differences in the relative protein abundance of ZO-1 were observed (*p* > 0.05).

## 4. Discussion

The level of intracellular ROS was maintained at a specific level, and any excessive ROS was typically eliminated by antioxidant enzymes under normal physiological conditions. Nevertheless, oxidative stress arises when the levels of ROS surpass the body’s antioxidant defense system, making it unable to remove them effectively [18]. Oxidative stress can damage proteins, lipids, and DNA, resulting in tissue injury and cell death, which can ultimately cause various diseases [19,20]. ROS production serves as a primary indicator of oxidative stress [21]. However, as the vital components in the antioxidant defense system, CAT, SOD, and GSH-Px can scavenge for ROS and reflect the body’s antioxidant capacity [22]. Whereas MDA is the final product of lipid peroxidation, the level of MDA reflects the degree of cell damage induced by oxidative stress. In the current research, the antioxidant capacity and underlying mechanisms of genistein were investigated through the use of the IPEC-J2 cell line as an in vitro cellular model. H_2_O_2_ was employed as the oxidant to induce oxidative stress as in previous research [23,24,25]. The findings of the current study indicated that H_2_O_2_ markedly decreased the IPEC-J2 cells’ viability, whereas pretreatment with genistein prior to H_2_O_2_ exposure markedly alleviated the decrease of cell viability caused by H_2_O_2_. To determine the antioxidant capacity of genistein, we analyzed ROS production, MDA levels, and the activities CAT, SOD, and GSH-Px. The results showed that genistein pretreatment decreased ROS levels, and enhanced CAT activity in H_2_O_2_-stimulated IPEC-J2 cells, indicating that genistein demonstrated protective properties and resists oxidative stress in IPEC-J2 cells. This result aligns with our previous study, in which daidzein (the aglycone form of the soybean isoflavones) significantly reduced MDA and ROS levels, and enhanced CAT activity when IPEC-J2 cells were stimulated by H_2_O_2_ [10]. Furthermore, our observations corresponded to other studies [26,27]. The isoflavonoid-enriched kudzu root extract recovered the downregulation of cell viability and alleviated the increase of ROS levels caused by rotenone in human umbilical vein endothelial cells (HUVECs) [26], and genistein pretreatment mitigated the decrease of SOD activity caused by oleic acid hydroperoxide in Caco-2 cells [27]. These results suggested that genistein had the potential to be used as an antioxidant against oxidative stress. In a future study, we will investigate whether dietary genistein improves the antioxidant capacity of pigs.

Demonstrations generally show that the Nrf2 signaling pathway protects against oxidative stress [28,29]. *Nrf2* was bound to Kelch-like ECH-associated protein 1 (*Keap1*) and primarily located in the cytoplasm in an inactive state under normal physiological conditions. Upon exposure to oxidative stress, *Nrf2* translocated from the cytoplasm to the nucleus after dissociating from *Keap1*, leading to the transcription of detoxifying enzymes [30,31]. In the current experiment, IPEC-J2 cells exposed to H_2_O_2_ had a significant lower gene expression of *Nrf2* and *CAT*, whereas pretreating cells with genistein before H_2_O_2_ exposure resulted in a significant increase in the gene expression of *Nrf2* and *CAT*. Furthermore, under the same conditions, the relative protein expression of Nrf2 correlated with the gene expression of *Nrf2* and *CAT*, indicating that genistein demonstrated protective properties and resisted oxidative stress in IPEC-J2 cells, and this mechanism may be associated with the Nrf2 signaling pathway. This observation was consistent with our previous study, in which daidzein exerted protective properties and resisted oxidative stress in IPEC-J2 cells by regulating *Nrf2* and its target gene expressions [10]. Similarly, in the research of Bai and Wang (2019), genistein protects against doxorubicin-induced cardiotoxic effects via activating the Nrf2 signaling pathway in mice models [11]. Miao et al. (2018) indicated that genistein could alleviate cerebral ischemia-induced oxidative stress injury in ovariectomized rats by promoting *Nrf2* and *NQO1* expression [32]. These findings demonstrated that genistein exerted an antioxidant capacity via the activation of the Nrf2 signaling pathway. However, the crucial role of the Nrf2 signaling pathway in genistein’s resistance to oxidative stress needs to be further confirmed by knocking out Nrf2 in our future research.

The intestinal epithelium serves a dual purpose: it facilitates the absorption of nutrients in addition to acting as a physical barrier preventing harmful pathogens, antigens, and toxins from the luminal environment permeating into the circulatory system [33]. Tight junctions, which are a key component of the intestinal physical barrier, have a strong association with intestinal permeability and play a vital role in maintaining gut health [34].

The structural composition of tight junctions consists of transmembrane proteins, such as zona occludens, occludin, and claudins [35], and ZO-1, occludin, and claudin 1 are three crucial tight junction proteins [36]. Previous studies have demonstrated that increased *ZO-1* and *occludin* expression is associated with reduced intestinal permeability in weaned piglets [37,38]. In the current study, IPEC-J2 cells exposed to H_2_O_2_ had a significantly lower expressions of occludin at the gene and protein levels and *ZO-1* at the gene level, whereas pretreating cells with genistein followed by H_2_O_2_ exposure reversed this change. Interestingly, we found H_2_O_2_ could reduce *ZO-1* expression at the gene level rather than at the protein level, indicating that H_2_O_2_ may predominantly regulate its expression at the transcriptional level. We speculate that the protective properties of genistein on the intestinal barrier may be linked to its antioxidant capacity, and further study needs to be carried out to explore the underlying mechanism of genistein affecting the intestinal barrier under oxidative stress. Collectively, our findings indicated that genistein has the potential to maintain intestinal barrier function under oxidative stress.

## 5. Conclusions

In summary, the current study revealed that genistein exerted protective properties and resists oxidative stress in IPEC-J2 cells, and the potential mechanism may be associated with the Nrf2 signaling pathway. The in vitro study that was performed will be replicated in vivo to confirm the results. Our findings can provide a theoretical basis for improving intestinal oxidative stress in piglets through nutritional interventions.

## Figures and Tables

**Table 1:** Primer sequences used for quantitative real-time PCR.

Gene	Forward (5′-3′)	Reverse (5′-3′)	Product Length, bp	Accession No.
*GAPDH*	GCTTGTCATCAATGGAAAGG	CATACGTAGCACCAGCATCA	86	NM_001206359.1
*SOD1*	GAAGACAGTGTTAGTAACGG	CAGCCTTGTGTATTATCTCC	93	NM_001190422.1
*CAT*	CCTGCAACGTTCTGTAAGGC	GCTTCATCTGGTCACTGGCT	72	NM_214301.2
*GPX1*	TCTCCAGTGTGTCGCAATGA	TCGATGGTCAGAAAGCGACG	104	NM_214201.1
*Nrf2*	GACCTTGGAGTAAGTCGAGA	GGAGTTGTTCTTGTCTTTCC	103	XM_005671981.3
*HO-1*	GAGAAGGCTTTAAGCTGGTG	GTTGTGCTCAATCTCCTCCT	74	NM_001004027.1
*NQO1*	GGACATCACAGGTAAACTGA	TATAAGCCAGAGCAGTCTCG	68	NM_001159613.1
*Occludin*	TCAGGTGCACCCTCCAGATT	TGGACTTTCAAGAGGCCTGG	112	NM_001163647.2
*ZO-1*	CGATCACTCCAGCATACAAT	CACTTGGCAGAAGATTGTGA	111	CV870309
*Claudin 1*	CCTCAATACAGGAGGGAAGC	CTCTCCCCACATTCGAGATGATT	76	NM_001244539.1

**Table 2:** Antibodies used in western blotting.

Antibody	Source	Dilution	Company	Cat#
Nrf2	Rabbit	1:1000	Abcam, Cambridge, UK	ab92946
ZO-1	Rabbit	1:1000	Thermo Fisher Scientific, Waltham, MA, USA	61-7300
Occludin	Rabbit	1:1000	Abcam, Cambridge, UK	ab31721
GAPDH	Rabbit	1:2000	Cell Signaling Technology, Danvers, MA, USA	2118

## Data Availability

The datasets used for the current study are available from the corresponding author upon reasonable request.

## Supplementary Materials

The following supporting information can be downloaded at: https://www.mdpi.com/article/10.3390/vetsci11040154/s1, Figures S1 and S2: Original image of Figure 6; Figures S3 and S4: Original image of Figure 7.
