# Supplementary material for: Genistein Alleviates Intestinal Oxidative Stress by Activating the Nrf2 Signaling Pathway in IPEC-J2 Cells
Source: Vet Sci. 2024 Mar 29;11(4):154. doi: 10.3390/vetsci11040154 (PMC11053601; doi:10.3390/vetsci11040154)
Supplement: Supplementary file 1 [file vetsci-11-00154-s001.zip › vetsci-2873108-supplementary.pdf]

Genistein Alleviates Intestinal Oxidative Stress by Activating the Nrf2 Signaling Pathway in IPEC-J2 Cells

Yanpin Li [1,†], Long Cai [1,†], Qingyue Bi [1,2], Wenjuan Sun [1], Yu Pi [1,*], Xianren Jiang [1] and Xilong Li [1,*]

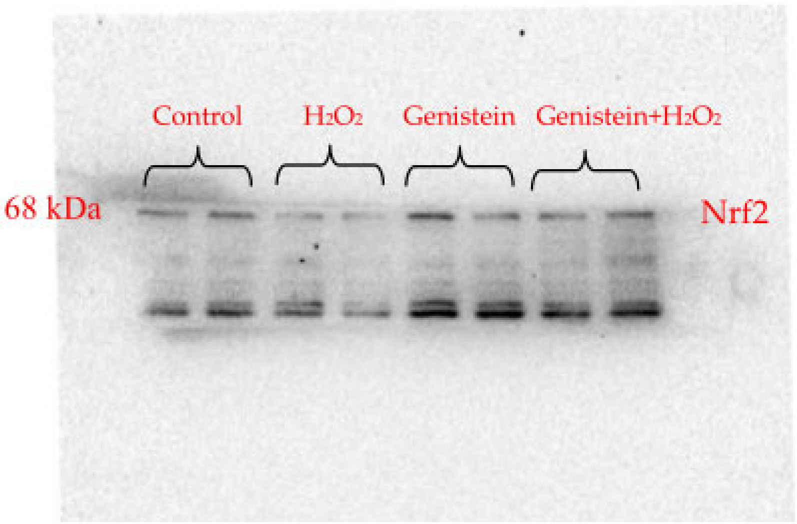

(**A**)

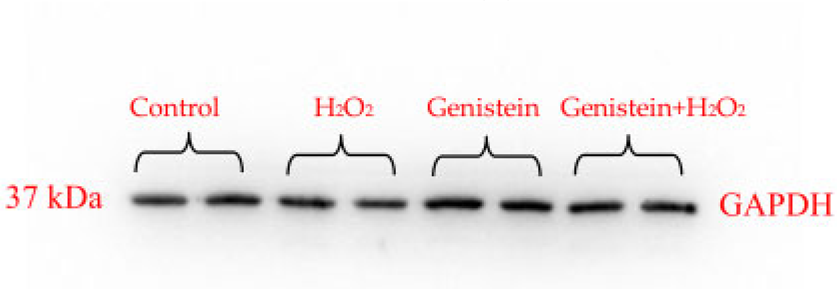

(**B**)

Figure S1. Original image of Figure 6. (**A**). Raw image of Nrf2 expression determined by Western blot. (**B**). Raw image of GAPDH expression determined by Western blot. According to Figure 1A and Figure 1B, the result for Nrf2 was Control (0.933 and 1.067), H2O2 (0.610 and 0.581), Genistein (1.276 and 0.879), Genistein+H2O2 (0.931 and 0.866), respectively.

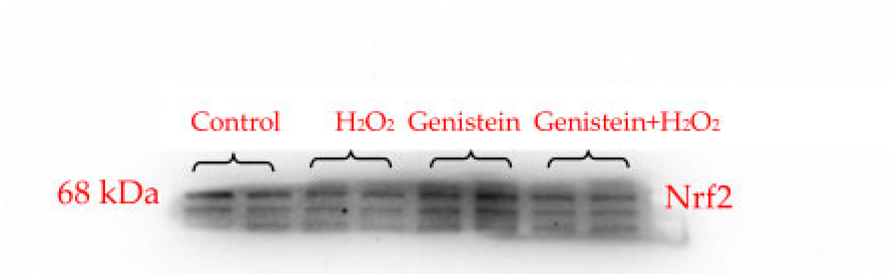

(**A**)

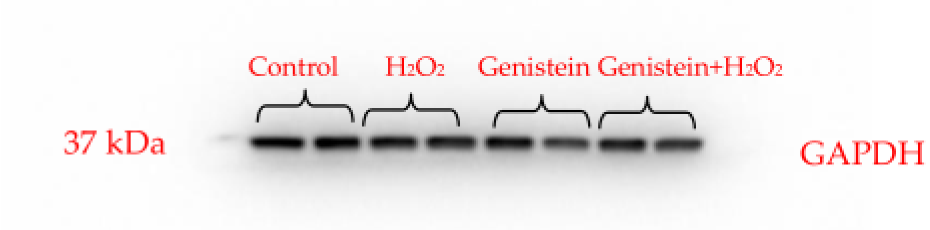

(**B**)

Figure S2. Original image of Figure 6. (**A**). Raw image of Nrf2 expression determined by Western blot. (**B**). Raw image of GAPDH expression determined by Western blot. According to Figure 2A and Figure 2B, the result for Nrf2 were Control (1.108 and 0.892), H2O2 (0.562 and 0.575), Genistein (0.926 and 1.255), Genistein+H2O2 (0.897 and 0.906), respectively.

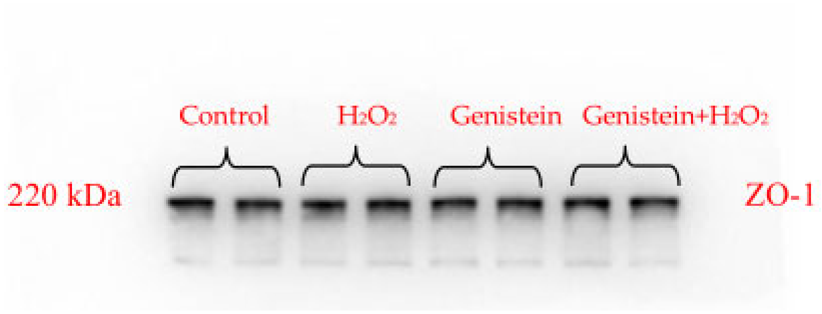

(**A**)

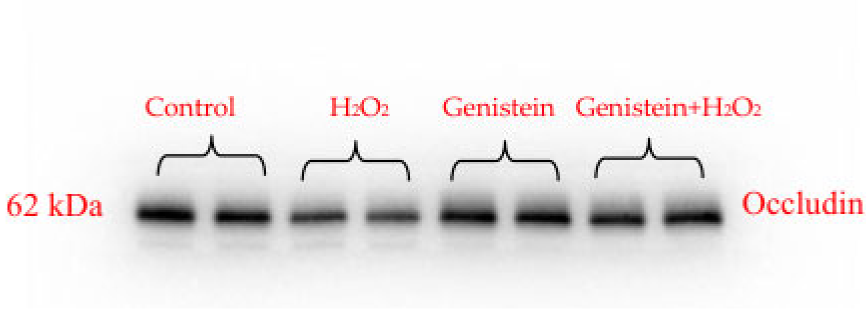

(**B**)

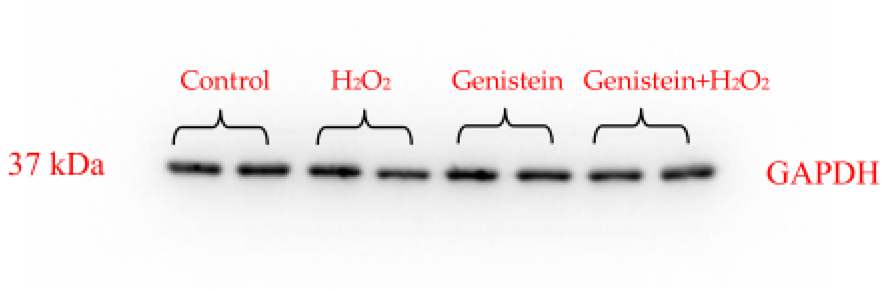

(**C**)

Figure S3:Original image of Figure 7. (**A**). Raw image of ZO-1 expression determined by Western blot. (**B**). Figure 3B. Raw image of Occludin expression determined by Western blot. (**C**) Raw image of GAPDH expression determined by Western blot. According to Figure 3A, Figure 3B and Figure 3C, the results for ZO-1 were Control (1.021 and 0.979), H2O2 (1.075 and 0.958), Genistein (0.929 and 1.150), Genistein+H2O2 (1.026 and 0.916), respectively, the results for occludin were Control (1.110 and 0.890), H2O2 (0.926 and 0.626), Genistein (1.110 and 0.898), Genistein+H2O2 (0.858 and 1.031), respectively.

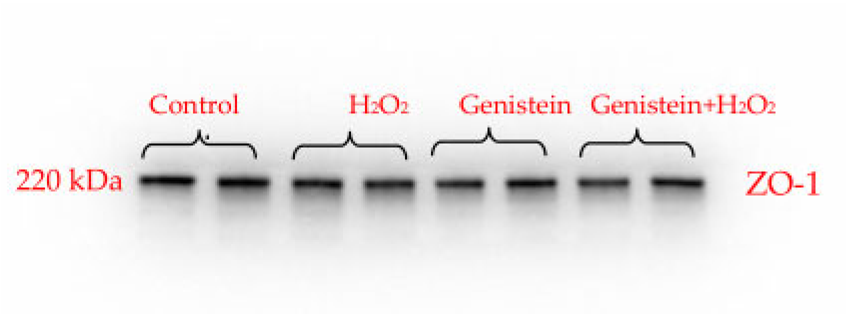

(**A**)

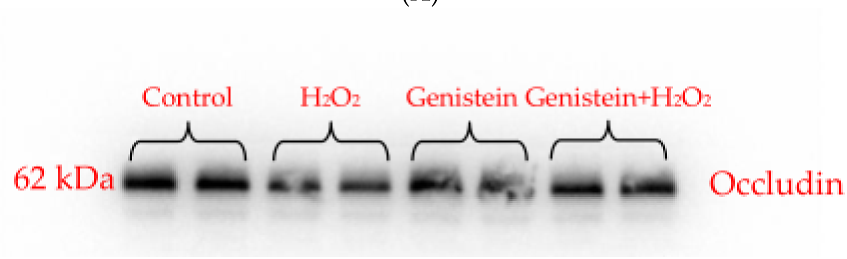

(**B**)

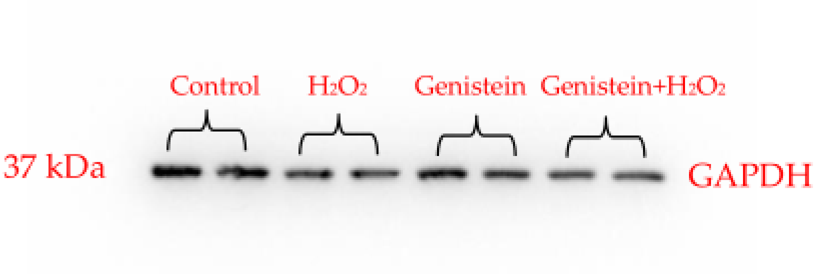

(**C**)

Figure S4. Original image of Figure 7. (**A**). Raw image of ZO-1 expression determined by Western blot. (**B**). Raw image of Occludin expression determined by Western blot. (**C**) Raw image of GAPDH expression determined by Western blot. According to Figure 4A, Figure 4B and Figure 4C, the results for ZO-1 were Control (1.059 and 0.941), H2O2 (1.233 and 0.911), Genistein (1.043 and 0.930), Genistein+H2O2 (1.068 and 0.977), respectively, the results for occludin were Control (1.096 and 0.904), H2O2 (0.701 and 0.832), Genistein (1.110 and 1.009), Genistein+H2O2 (1.048and 1.045), respectively.
